# STDD: Short-Term Depression Detection with Passive Sensing

**DOI:** 10.3390/s20051396

**Published:** 2020-03-04

**Authors:** Nematjon Narziev, Hwarang Goh, Kobiljon Toshnazarov, Seung Ah Lee, Kyong-Mee Chung, Youngtae Noh

**Affiliations:** 1 Department of Computer Science and Information Engineering, Inha University, Incheon 22212, Korea; nnarziev@nsl.inha.ac.kr (N.N.); hrgoh@nsl.inha.ac.kr (H.G.); kobiljon@nsl.inha.ac.kr (K.T.); 2 Department of Psychology, Yonsei University, Seoul 03722, Korea; foryou@yonsei.ac.kr

**Keywords:** depression tracking, short-term detection, passive sensing, EMA

## Abstract

It has recently been reported that identifying the depression severity of a person requires involvement of mental health professionals who use traditional methods like interviews and self-reports, which results in spending time and money. In this work we made solid contributions on short-term depression detection using every-day mobile devices. To improve the accuracy of depression detection, we extracted five factors influencing depression (symptom clusters) from the DSM-5 (Diagnostic and Statistical Manual of Mental Disorders), namely, *physical activity*, *mood*, *social activity*, *sleep*, and *food intake* and extracted features related to each symptom cluster from mobile devices’ sensors. We conducted an experiment, where we recruited 20 participants from four different depression groups based on PHQ-9 (the Patient Health Questionnaire-9, the 9-item depression module from the full PHQ), which are *normal*, *mildly depressed*, *moderately depressed*, and *severely depressed* and built a machine learning model for automatic classification of depression category in a short period of time. To achieve the aim of short-term depression classification, we developed Short-Term Depression Detector (STDD), a framework that consisted of a smartphone and a wearable device that constantly reported the metrics (sensor data and self-reports) to perform depression group classification. The result of this pilot study revealed high correlations between participants` Ecological Momentary Assessment (EMA) self-reports and passive sensing (sensor data) in physical activity, mood, and sleep levels; STDD demonstrated the feasibility of group classification with an accuracy of 96.00% (standard deviation (SD) = 2.76).

## 1. Introduction

Depression has affected approximately 25% of the world’s population at least once in their lifetime, and around 7% of the population annually [1]. People with depression experience not only psychological difficulties but also physical symptoms and show higher suicide rates (200% compared to general population), resulting in higher medical costs [2,3]. Therefore, depression has become a salient public health issue in this era. However, assessing symptoms of depression, and their severity is not straightforward. The most widely used methods among mental health professionals are interviews and self-report questionnaires. Unfortunately, these methods are time-consuming, expensive, and often require the involvement of professionals. 

To cope with this, passive sensing with smartphone and wearables (devices that track daily activities and routines) are part of a promising and rapidly growing area in pervasive health care. Smartphones and wearables are capable of capturing multiple dimensions of human behavior, encompassing physical, mental, and social aspects of well-being [4,5]. An additional benefit is that passive sensing can provide short-term detection of certain conditions and help with the prevention and long-term management of symptoms. Therefore, helping patients identify patterns in their behavior and recognizing factors that impact their mental state would provide them with greater insight into the nature of the disease, which would in turn help them to cope better. In this work, we deliberately extracted five symptom clusters for depression from the Diagnostic and Statistical Manual of Mental Disorders (DSM-5) [6], namely, physical activity, mood, social activity, sleep, and food intake, to improve the accuracy of depression diagnosis and detection. Furthermore, we developed Android and smartwatch applications to collect information from related embedded sensors for the five symptom clusters. To achieve the aim of depression category detection in a short period of time, we categorized participants into four groups, namely, normal, mildly depressed, moderately depressed, and severely depressed and trained a machine learning model using passive sensing and Ecological Momentary Assessment (EMA) data. We believe that our framework can be a great baseline for studies related to interventions that can be tailored in a timely manner for people from each category of depression.

We designed Short-Term Depression Detector (STDD), a framework that can track the five depression symptom clusters from collected EMA and passive sensing data, and conducted a four-week pilot study with 20 participants to address the following questions: 

• How similar are the levels of each symptom cluster classified using passive sensing with those answered by participants using EMA? 

• Is it possible to accurately classify different depression categories by using passive sensing data related to each symptom cluster along with EMA answers over a short period of time? 

In this work, we have made the following concrete contributions: 

• We deliberately extracted five depression symptom clusters from DSM-5, namely, physical activity, mood, social activity, sleep, and food intake, to improve the accuracy of depression diagnosis and detection and worked with information collected from passive sensing technology, such as mobile and wearable devices (smartphone and smartwatch) (Section 3.2) 

• We proposed STDD, a framework that can track the five depression symptom clusters from collected passive sensing data and performed group classification for the future intervention study. STDD consists of smartphone and smartwatch agents that gather data, as well as a cloud management platform, which contains a dashboard to easily track data quality and participants’ negative behaviors such as turning off sensors and forgetting to self-report. (Section 4) 

• We conducted a pilot study with 20 participants to extract a group classification model (normal, mild, moderate, and severe) and to verify its feasibility with PHQ-9. The derived model achieved an accuracy of 96.00% (standard deviation 2.76). (Section 5)

## 2. Related Work

Recently, there have been a number of studies on using passive sensing automated smartphones and wearables to predict mental health and well-being. In previous studies [7,8,9,10,11,12], smartphone applications were developed to collect data for assessing the mental health status of college students and adults. The studies found significant correlations between low level sensor data (i.e., activity, conversation, and mobility), depression severity, perceived stress level, and academic performance (i.e., GPA scores) [7] have been reported. In another branch of study [8], self-ratings of mood were collected from students five times per day using the Ecological Momentary Assessments (EMAs, [13]) while sensor data were continuously recorded. The authors compared various classification methods for predicting short-term mood from mobile phone data, such as the number of calls and messages. In [10], an app-based intervention was designed to reduce depressive symptoms, and the usability and efficacy of this intervention were also examined. 

Another area of research focuses more on predicting clinical depression and its severity using a variety of sensing data. Outcomes from self-report questionnaires, including the Patient Health Questionnaire-9 (PHQ-9) [14], are commonly used as ground truth (GT) for measuring severity of depressive symptom. Saeb et al. [15] used a modular system Purple [16] to extract significant features related to depressive symptom severity from GPS and phone usage data. Their studies defined features such as location variance, entropy, time spent at home, circadian movement, transition time, and frequency and duration of phone usage. The results of their analysis showed that six of the 10 features were significantly correlated with the level of depression that was evaluated by the PHQ-9 score. 

FINE [17] is an early-stage Android app that collects self-reports (mood diary and PHQ-9 score) and sensor data, which is then categorized into three groups: smartphone usage pattern (screen on/off, time on screen, and frequently used apps), communication (incoming/outgoing/missed calls and SMS), and movement (triaxial acceleration and location). It is worth noting that parameters correlated with depression, and the features, chosen by the FINE study, were in accordance with previous research, such as [15]. The small number of participants (n = 4) and short period of data collection (one week) were pointed out as limitations, in addition to some security and sampling problems in the process of data collection. 

As an early attempt to exploit the potential of mobile interventions for depression, Mobilyze! [5] applied machine learning techniques to the task of monitoring patients’ status and circumstances and delivering ecological momentary intervention. Mobilyze! predicted categorical contextual states (such as location, friends in the immediate vicinity, or alone) with good accuracy (the mean ranged approximately from 60% to 91%), but the accuracy for predicting mood states, assessed with self-reported scales, needed further examination. In study [18], the authors developed a smartphone application iHOPE for the daily monitoring of mood and other depression-related indicators, including sleep patterns and cognitive performance using EMA. They collected EMA data from 59 patients, and the results suggested that smartphone-based EMA was highly feasible. In addition, smartphone applications and wearable sensors, for monitoring mood-related parameters, have been utilized for individuals with bipolar disorder and suicidality [19,20,21,22,23,24], which are mostly characterized by manic and depressive episodes. For example, the PRIORI app [20] collected conversational data from which the authors extracted rhythm features, given that individuals with bipolar disorder exhibit changes in the rhythm of their speech. The study suggested that, through certain pre-processing, feature extraction, and data modeling techniques, it was possible to mitigate the effects of differing amounts of clipping, loudness, and noise.

Table 1 summarizes the aforementioned works of depression tracking and clearly defines usage of passive sensing, EMA, and interventions in the literature.

## 3. Study Design

**Figure 1:**
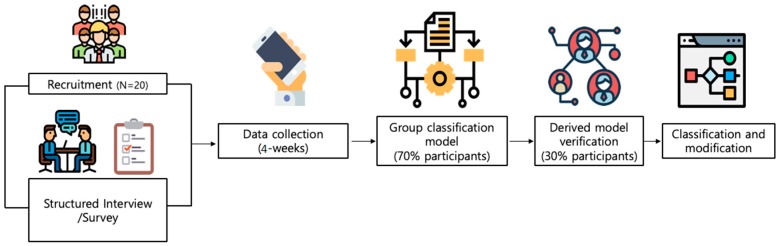
Study procedure of depression group classification: 20 participants for four weeks.

Figure 1 illustrates our overall study procedure. In the beginning, we recruited participants from online social communities and carefully selected 21 people based on their depression severities (i.e., 5 normal, 6 mild, 6 moderate, 4 severe). We collected six EMAs per day (once each 3 h, starting from 7 a.m.) and passive sensing data from Android smartphone and smartwatch (Galaxy S3) sensors for four weeks. To derive a group classification model via machine learning, we trained and tested 70% and 30% of participants, respectively. We present results and our findings in results section.

### 3.1. Entry and Exit

**Figure 2:**
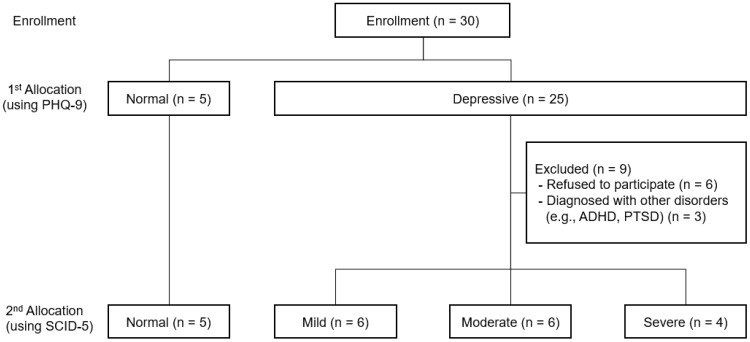
Consort flow diagram: recruitment phase.

As outlined in Figure 2, participants were recruited from online social communities for college students. The PHQ-9, which is a brief and self-reported screening tool for depression, was first administered to every student who wanted to participate in this study via Qualtrics [25], an online survey platform. Participants were classified into non-depressive (normal) group and depressive group according to the PHQ-9 cut-off score, and depressive group was classified into three groups (mild, moderate, and severe/clinical) again according to the results of the Structured Clinical Interview for DSM-5 (SCID-5). Although the SCID-5 is a well-established diagnostic assessment tool, it takes an average of 1.5 to 2 h to administer. Therefore, in order to save time and effort in recruiting almost equal number of participants across four groups using the clinical interview, the PHQ-9 was used in the first step of screening. The detailed information about group classification is described in the next section. In addition, the Beck Depression Inventory-II (BDI-II) [26] and State-Trait Anxiety Inventory (STAI) [27] were administered, to confirm the severity of depressive symptoms and to measure anxiety symptoms, respectively. The level of anxiety measured by STAI was used as a supplementary information about participants, since it has been consistently reported that individuals with comorbid depression and anxiety have more severe symptoms [28]. EMA and passive sensing data were collected after informed consent was obtained from all participants. At the exit stage, we administered the self-reported questionnaires (PHQ-9, BDI-II, and STAI), again to measure changes in symptom severity, and a short interview about the overall life pattern, mood changes, and user experience during the data collection phase. This study was approved by the Institutional Review Board of Yonsei University (No. 7001988-201905-HR-606-03).

### 3.2. Depression Gauge: 5 Symptom Clusters

**Figure 3:**
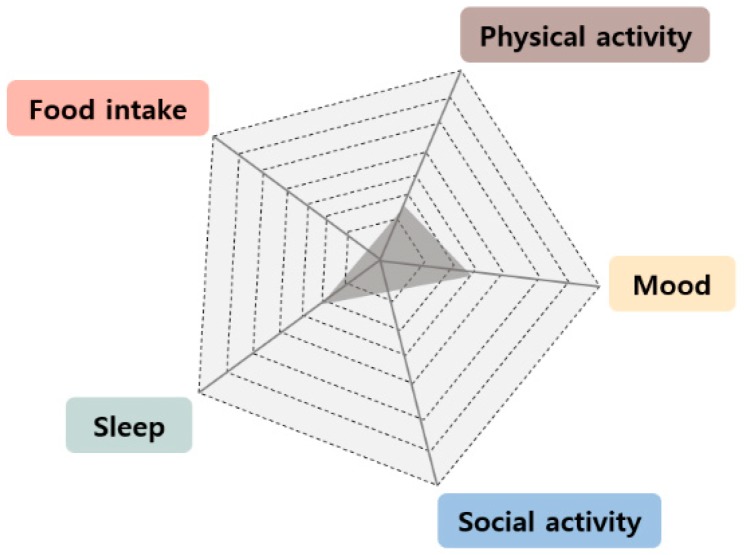
Radar chart (pentagon shape): depression symptom clusters.

Figure 3 depicts a radar chart of the five depression symptom clusters. According to the DSM-5 criteria, one of the primary symptoms should be either a depressed mood or loss of interest or pleasure. The secondary symptoms of depressive disorder include appetite or weight changes, sleep difficulties, psycho-motor agitation or retardation, fatigue or loss of energy, diminished ability to think or concentrate, feelings of worthlessness or excessive guilt, and suicidality. We extracted these five factors as indicators of depression severity, according to the DSM-5 questionnaire, namely, mood, physical activity, sleep, social activity, and food intake. Food intake was chosen because we concluded that food intake could be one of the measurable behaviors to assess appetite or weight changes based on findings that food intake was changed when people became depressed [29,30].

### 3.3. Group Classification

We classified participants into four groups as following: (i.e., normal), mildly depressed, moderately depressed, and severely depressed (i.e., clinically depressed). The normal group included participants who reported scores of 0–9 on the PHQ-9 scale and neither had a history of psychological disorders nor presented depressive episodes during the clinical interview. Among the participants who reported PHQ-9 scores greater than 10, which is recommended as the optimal cut-off score for diagnosing depression [31,32,33], participants in (full or partial) remission from previous depressive episodes or who did not satisfy the full criteria for a current depressive episode with no specific diagnosis at the clinical interview were assigned to the mildly depressed group. The moderately depressed group included participants who partially met the criteria for major depressive disorder and satisfied the criteria for other depressive disorders (e.g., persistent depressive disorder). The severely depressed group consisted of participants with a confirmed diagnosis of major depressive disorder from the clinical interview. The mean score of PHQ-9 of four groups were 2.5, 14.2, 15, and 15 for normal, mildly depressed, moderately depressed, and severely depressed group, respectively. The mean BDI and STAI scores for normal group were 5.25 and 65, 20.8 and 102.2 for mildly depressed, 25 and 110 for moderately depressed, and 26.8 and 111.3 for severely depressed group. 

### 3.4. Data Collection and Privacy

We collected participants’ data from both smartphone and smartwatch applications that were passively reporting sensor data. EMA questionnaires were delivered according to the schedule (six times per day) through notifications to the participants’ smartphones. More details on the data are discussed in the following section. Because STDD collected sensor data from smartphone and smartwatch, the privacy of the participants was a major concern. We generated hash IDs to randomize participants’ identities and uniquely access all collected sensor data for each participant without any identification. All collected sensor information, including call logs and app usage, were received and uploaded via SSL connection to the STDD cloud.

## 4. System Architecture and Passive Sensing

**Figure 4:**
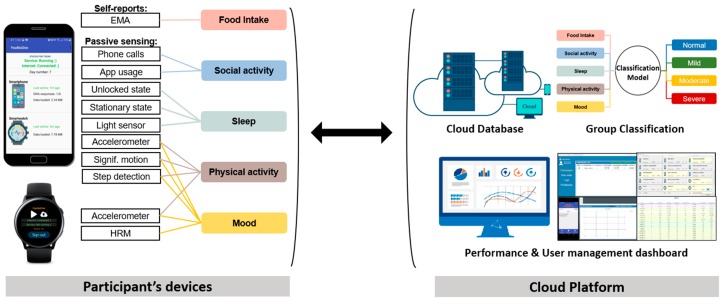
Short-Term Depression Detector (STDD) architecture: smartphone and smartwatch apps and cloud platform with dashboard.

Our sensing system consists of two main components (see Figure 4), namely, participant’s devices and cloud platform. For the first component, to collect sensor data and EMA self-reports from participants, we developed smartphone (Android) and smartwatch (Gear S3 with Tizen OS) applications on Android Studio and Visual Studio IEDs, respectively. At the beginning of the study, we asked participants to register using smartphone app and use the same credentials in smartwatch app to sign in and start using the applications without any restrictions. Both devices were passively collecting sensor data, while self-report questions were delivered to the participants at the scheduled times through notifications that were sent to their smartphones. 

**Figure 5:**
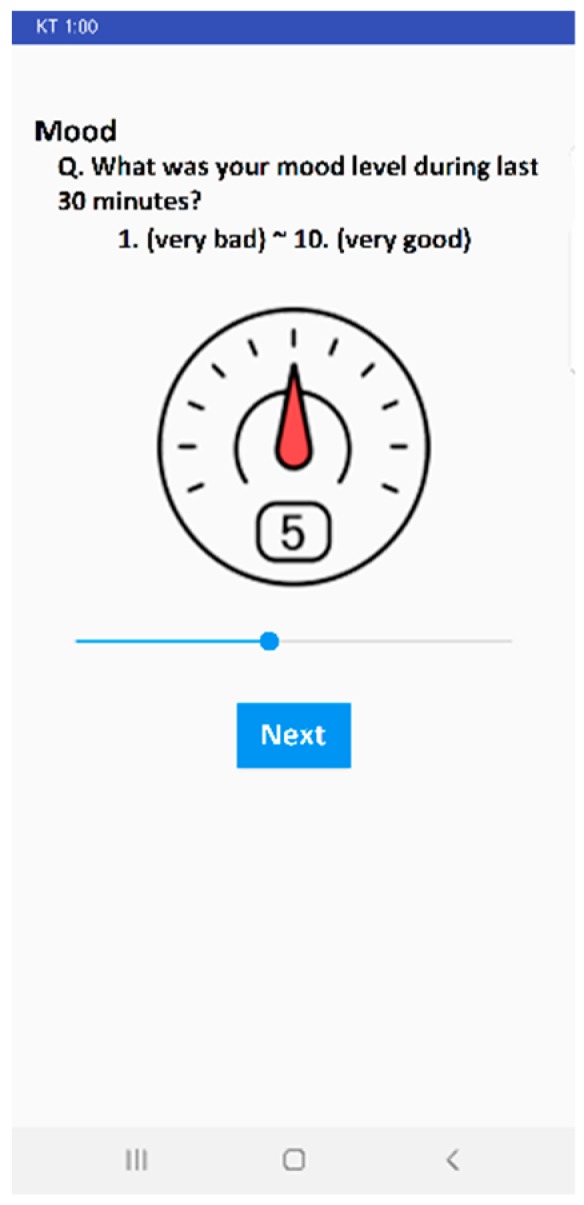
An Ecological Momentary Assessment (EMA) snapshot on mood.

We used the in situ EMA [13] to capture additional human behavior, beyond what the surveys and passive sensing provided, and used it as the subjective ground truth (GT). The participants were prompted to answer a short survey consisting of five questions (one for each depression symptom cluster); this was scheduled to appear 6 times per day (7:00 a.m., 10:00 a.m., 1:00 p.m., 4:00 p.m., 7:00 p.m., and 10:00 p.m.). For every question, they were provided a scale of ordinals from 1 to 10 representing the level of corresponding symptom cluster. Figure 5 shows a snapshot of the EMA question on the mood. For passive sensing data collection, we used multiple data sources from the mobile devices. We, then, matched data sources with five symptom clusters (shown in Figure 3) to represent each cluster with one or multiple data sources. The detailed list of features extracted from data sources to infer every symptom cluster is described in further subsections. All passive sensing data collected from the mobile devices were stored locally as separate CSV files and transmitted to our secure cloud server whenever participants were connected to the internet via Wi-Fi.

The second component of STDD, cloud platform equipped with the Django framework [34], received the files and processed/stored them in the PostgreSQL database. To minimize the data loss, we created a simple dashboard to easily monitor participants’ data provision performance statistics and contacted them through SMS and phone calls if any misleading data provision was noticed.

### 4.1. Observational Study and Depression Group Classification

**Figure 6:**
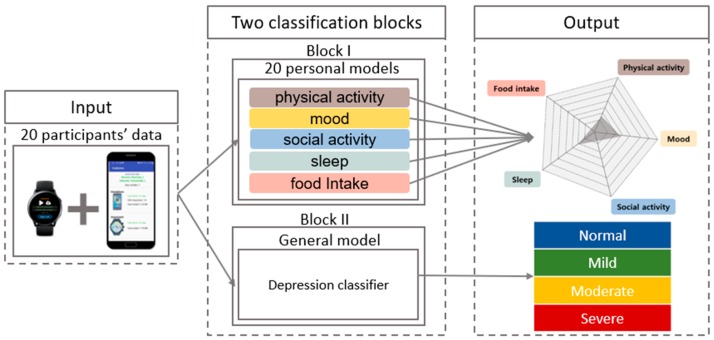
Flow diagram of depression group classification approach.

In this study, we developed two classification blocks (Figure 6), one for observational purposes and the second for our goal of depression category classification. The first block was created with the aim of investigating where to classify the level of each symptom cluster using passive sensing data and compared it to EMA data. As depicted in Figure 6, block-I consists of 20 personal models where each model takes features, derived from the mobile devices of the corresponding participant as an input and outputs level of each symptom cluster. Block-II processes the same feature list as input and outputs the depression category. These blocks are further explained in the following paragraphs separately. In both blocks, we applied two models in WEKA [35] for classification, namely, Support Vector Machines (SVM) and Random Forests (RF). SVM generates hyperplanes in multidimensional space that separate the data into classes in a best way. Our implementation of SVM used a gaussian radial basis function for the classification task. RF consists of a large number of individual decision trees that operate as an ensemble. Each individual tree in the RF spits out a class prediction, and the class with the most votes becomes our model’s prediction.

#### 4.1.1. Block for Observational Study

This study was conducted for an initial proof-of-concept with only 20 participants. Therefore, prior to depression group classification based on five symptom clusters, we developed the first block where we built personalized models for each participant to detect their symptom levels using passive sensing data. We wanted to compare predictions derived using sensors’ data to EMA data and verify the possibility of symptom classification using passive sensing. In the results section, we showed the comparison between passive sensing classification and EMA responses. The following paragraphs describe what sensors were used and which features were extracted for classification of the level in each symptom cluster. Physical activity and mood levels were detected using machine learning algorithms, while social activity, sleep, and food intake levels were classified using other methods described below.

*Physical activity:* We collected accelerometer, significant motion, and step count data from the Android smartphone and smartwatch (Gear S3 Frontier) to infer physical activity level of participants. Our feature extraction approach was similar to that used for the activity recognition function from smartphone accelerometer data [36]. The feature extraction was performed on fixed length windows of 50 samples (1 s) with 50% overlap. For each window we extracted a total of 17 features [36], which were (1) mean, (2) standard deviation, (3) maximum, (4) minimum, (5) energy, (6) kurtosis, (7) skewness, (8) root mean square, (9) root sum square, (10) sum, (11) sum of absolute values, (12) mean of absolute values, (13) range, (14) median, (15) upper quartile, (16) lower quartile, and (17) median absolute deviation. These 17 features were extracted from each of the three axes of the raw accelerometer data, resulting in 51 features. The total number of accelerometer features was 51 × 2 = 102, because the raw data was extracted from both smartwatch and smartphone. Each window was treated as an independent sample (feature vector). Moreover, we computed the total number of steps and significant motion sensor triggers from Android phone. Table 2 shows a list of features used for detecting physical activity and mood levels.

To characterize each EMA answer, we took the features from the previous 30 min for each EMA. According to our feature extraction method with sliding window as explained above, we had a large number of feature vectors from accelerometer (30 × 60 × 50% overlap = 3600 samples). Our approach was to duplicate EMA response for physical activity level across all data samples, since we believed that this way, machine learning model could better learn all possible physical activity variations that map to the same label (physical activity level). We divided the data into 20 datasets, that is, one for each participant and built personal models with the features described above. Our models underwent 10-fold cross-validation, that was repeated 10 times using the final dataset split into 70% to 30% train-test sets. After comparing SVM with RF classification algorithm, we finally used the RF classifier and achieved more than 90% average accuracy in each accuracy measure across all 20 personalized models, which are shown in Section 5 with precision, recall, F-measure, and true positive rate.

*Mood:* For the classification of mood level, we used 104 physical activity features with additional heart rate monitor (HRM) sensor features as shown in Table 2 and used the same classification method as described for physical activity above. Mean heart rate and standard deviation features were extracted from HRM sensor, which are reported in [37] as key supplementary information for the mood classification. The EMA results for mood were used as ground-truth data. Similarly, we built models for each participant for mood level prediction and used 70% of the overall data to train each model and verified accuracy using remaining 30% test data. The average accuracy measures on mood level classification across all 20 personalized models are presented in Section 5.

*Sleep:* We implemented a sleep classifier based on checking following conditions: when smartphone was static, not being used, and the environment was dark (with light intensity of 0–10 lux extracted from light sensor). Those conditions were tracked during the potential hours of sleep (10:00 p.m.–10:00 a.m.). Finally, our classifier identified the longest non-usage period as the sleep duration of the day. To handle scaling, we defined the baseline to be 7 h (the recommended duration of sleep) and scored the sleep duration of participants based on how far they deviated from the baseline (i.e., 3 h and 11 h are scored as 1). 

*Social activity:* We collected the number of incoming and outgoing phone calls, call duration, and social communication app usages. We adopted the same formula of weighted sum as in [21]. The “social incoming” and “social outgoing” features were generated from the number of incoming and outgoing calls and their duration. We could not collect the number of incoming and outgoing messages as in [21], because people in Korea mainly rely on a social communication app called KaKaoTalk [38], from which we could not extract information directly. Therefore, we counted the frequency of opening other social apps (i.e., Twitter, Facebook, etc.) and their durations. To balance the weight of the features, we multiplied them by a constant value (10). The value could be calculated using aggregate functions or probability methods, but we chose a constant value for simplicity; because this formula is the same for all data instances, the results remained consistent. 

*Food intake:* In this study, we only collected food intake indicators only from self-reports because our aim was to minimize obtrusiveness to participants (i.e., taking a picture). Therefore, we added questions on food intake using a Likert scale on every EMA, but we did not track participants’ weight changes in this study.

#### 4.1.2. Block for Depression Category Classification

After our observations on symptom level classification using passive sensing, we built a general model for all participants to classify their depression groups. We utilized all the features used to classify each symptom cluster level along with EMA responses from participants to build a dataset for training our general model. However, we realized that this approach was not accurate enough with a small number of participants and left it to utilize during the large-scale data collection experiment in our upcoming study with more than 30 participants in each depression group. Therefore, in this study, we decided to utilize the classified levels outcomes for each symptom cluster from personalized models as input for our depression classification model. In Section 6 we described the limitation of this approach. The final feature vector for depression classification consisted of five elements, which are the classified levels for physical activity, social activity, mood, sleep, and food intake, separately. The level of food intake was not predicted from passive sensing but was directly taken from EMA responses. Since for every 30-min period we had one sample with five symptom cluster levels, the total number of samples was supposed to be around 3600 (6 EMAs × 30 days × 20 participants). However, in this study, we faced a problem of data loss and EMA skips by participants. Therefore, we only used 2046 data samples that were available in our dataset for classification and accuracy validation. For the outcome variable, which is depression category, we considered that the categories of participants remained stable, which is widely accepted in the psychology domain. We assumed that each depression group had its own daily symptom cluster variation patterns which were not clearly seen. Therefore, for 30 days of study, we decided to link all the symptom variations of a participant with his depression group and trained ML model to make it learn how to correctly associate any symptom cluster variation with the most probable depression group, hence we put the same depression group (outcome variable) across all of the samples of the same person. Finally, we used 70% of 2046 data samples as training set and the remaining 30% for test set. After applying classifiers of depression category using WEKA [35], we verified that the best performing model RF with the number of decision trees to grow to 100. In Section 5, we described the accuracy of the model. 

## 5. Results

We conducted a pilot field study as outlined in Figure 1. After the study, we extracted the response rates for EMA from final dataset. Table 3 represents the averaged response rates of all participants. Each EMA check-in point is time when STDD application prompted each participant to answer an EMA question. It is also clearly shown that the response rate at 7 a.m. is quite lower than other hours.

**Figure 7:**
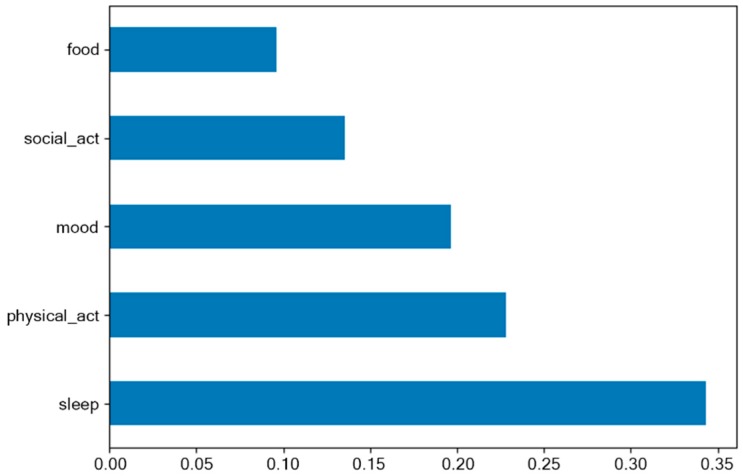
Distribution of feature importance for depression classification model.

To address the model’s interpretability, we examined the information gain of the features, that is, how important was each feature to perform a classification. In Figure 7 we presented the feature importance for depression classifier. According to our results, the highest contribution among other features had a sleep level with 34% contribution to the model performance.

**Figure 8:**
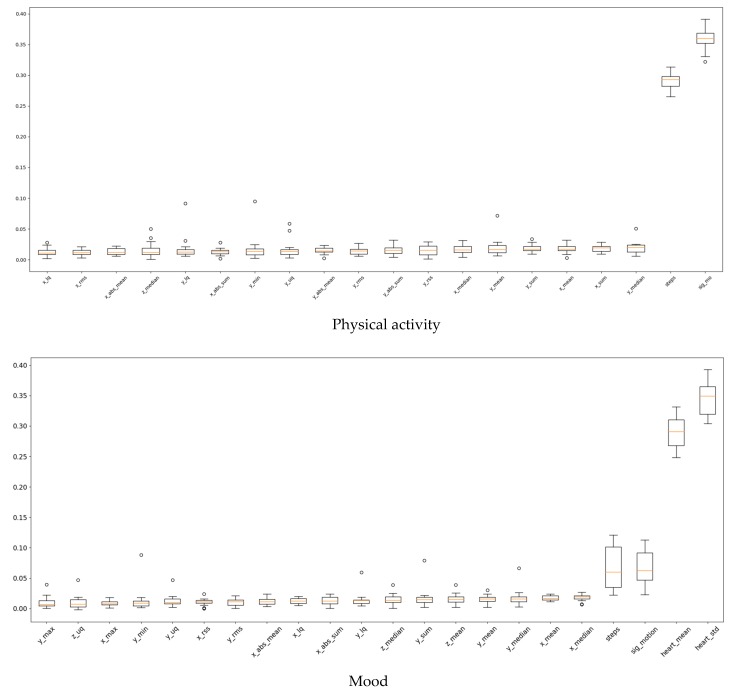
Distribution of feature importance per feature for all personal models for classification of physical activity and mood levels.

We also presented feature importance results for symptom level classification in Figure 8. Because we built personalized models for classification of mood and physical activity levels, features that could be important for one participant might be less important for a different participant. To show this, we plotted the distribution of feature importance values for each feature across all the participants using boxplots below. We included only the top 30 features (sorted by their median) for visibility, with the trend of remaining features being about the same. According to results, for physical activity level classification, significant motion and step features were highly important, while for the level classification of mood heart rate sensor features were dominant.

**Figure 9:**
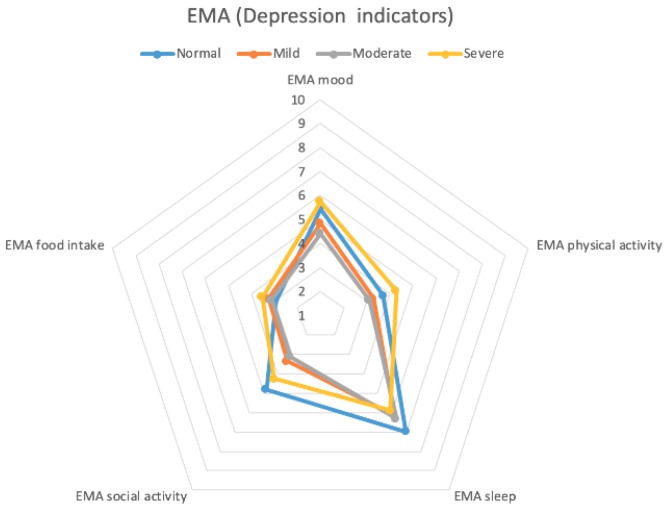
Averaged depression indicators with EMA.

**Figure 10:**
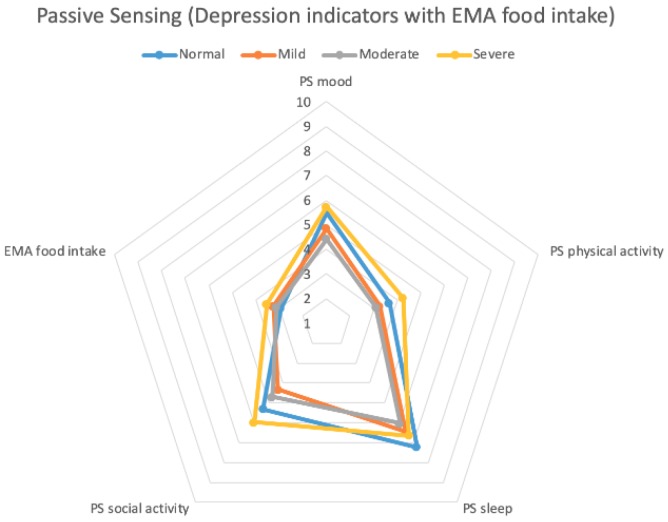
Averaged depression indicators with passive sensing.

Figure 9 and Figure 10 show radar charts of averaged symptom clusters levels by EMA and passive sensing data. These two charts show similar behaviors. We collected food intake indicators only from the EMA, because of the need for passive sensing to be unobtrusive. For the mood and physical activity indicators, the severe group reported slightly larger values than the normal group, whereas the normal group showed slightly larger values than the severe group in social activity and sleep. The mild group showed smaller values in the five indicators than the normal and severe groups but larger values than the moderate group. Our findings show that the size of the five indicators becomes smaller from the normal to mild group and from the mild to the moderate group. However, the severe group shows counter-intuitive results. We found that one of the four participants in the severe group suffered from bipolar disorder (particularly in the mania phase), so this participant showed a large value in all five symptom clusters.

**Figure 11:**
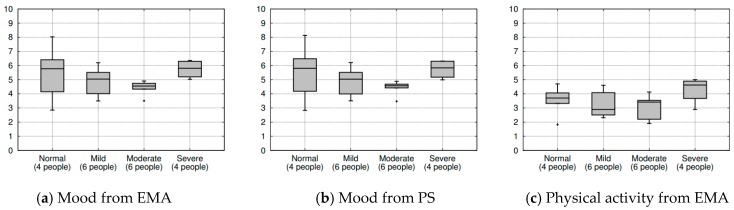

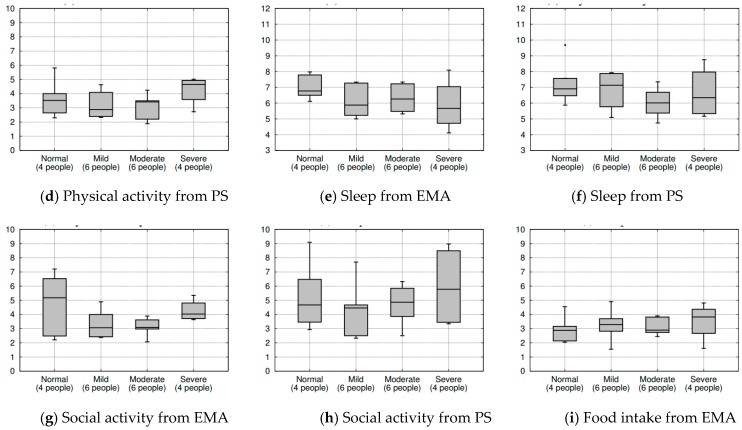
Box plots of EMA and passive sensing on five elements as a function of four participant groups: (**a**,**b**) mood; (**c**,**d**) physical activity; (**e**,**f**) sleep; (**g**,**h**) social activity; (**i**) food intake.

Figure 11 shows box plots of all self-reported EMA and automated passive sensing data on five elements (i.e., mood, physical activity, sleep, social activity, and food intake). We used a 10-point Likert scale (y-axis) as a function for the four participant groups divided by depression category (x-axis). We experienced data loss caused by technical difficulties affecting one participant in the mild group. Therefore, we report data from 20 participants.

Figure 11a,b shows the mood distribution of the four groups. There was a distinct downward trend from the normal group, via the mild group, to the moderate group; however, the severe group showed a behavior similar to the normal group. We expected that this might have been influenced by bipolar disorder (i.e., the mania phase) behaviors or an attempt to overcompensate in the severe depression group. 

Figure 11c,d shows the physical activity from EMA and passive sensing data, respectively. We observed similar trends to mood. However, the values were slightly larger in the severe group. This also demonstrates the limitation of having small sample sizes, because one outlier can severely affect the overall behavioral results. We expect that increasing the number of participants will solve the problem and better show the overall trends.

Figure 11e,f shows the sleep data from EMA and passive sensing data, respectively. In both figures, the sleep duration does not seem to vary much among the groups, but it does seem to gradually decrease from the normal group to the severe group. These two figures show an interesting point. In the EMA, participants believe that they sleep more hours from a subjective viewpoint, but they actually sleep less from an objective viewpoint (i.e., the passive sensing data). Figure 11g,h shows the social activity from EMA and passive sensing data, respectively. 

Figure 11i shows the food intake from the EMA data only. These results show little difference between the normal, mild, and moderate groups, whereas participants in the severe group showed larger values of food intake. We expected that depression might increase the food intake of the participant.

The average accuracy measures (precision, recall, F-measure, and true positive rate) on mood and physical activity level classification across all 20 personalized models are presented in Table 4.

Table 5 shows the results of group classification in each depression group. For fairness, we tested our classifier with the same number of data samples (150 samples in this setting) from 30% of remaining dataset to verify the performance. As shown in the table, we achieved overall accuracy of 96.0% (SD = 2.76).

## 6. Discussion and Limitations

In this study, we proposed the development of STDD to prove the possibility of a depression group classification system and deliver design implications. Nevertheless, we discuss here the limitations of this study and the design implications for future apps of this type, such as mood detection with passive sensing, EMA collection, and physical activity monitoring. 

In this pilot study, we set a 30 min limit for each EMA deadline to make the self-reported EMA vivid (rather than retrospective). However, this design choice caused the participants to easily miss the deadlines, and our final dataset was quite limited, particularly at the 7 a.m. EMA check-in point (possibly because of late wake-up or rushing to work or school) as shown in Table 3. In the next study, we will extend the deadline to immediately before the next EMA and display a progress bar, to encourage participants to complete the EMAs more actively.

For the physical activity and mood detection, we used accelerometers and heart rate monitor sensors. To deal with scaling, we extracted 51 sensor features and trained them with each participant’s EMA reports (30 min before each EMA). However, this personalized training model caused physical activity and mood values from passive sensing to be the same as those from the EMA. This renders the group classification model redundant to the EMA results for physical activity and mood. To make the passive sensing result a distinct feature for group classification, we plan to use Google activity detection with the timekeeping. For mood, Canzian et al. reported that mobility patterns from GPS traces can be used to extract people’s mood [39]. As another direction, voice features (such as pitch, frequency of voice, and speaking rate) can be used for mood detection. This will provide more information to improve the group classification model. Finally, our approach to the group classification using the output from personalized models suffered from overfitting. In other words, since each personalized model had unique parameters that were fitted for each participant, classification of depression severity relied on those parameters.

Although our scoring method for sleep level was accurate according to EMA responses (with Pearson Correlation Coefficient of r = 0.68), we still were not able to detect whether participants woke up at night until they did not start using their mobile devices. We also computed the PCC to find the correlation between EMA responses and classification results using passive sensing data on social activity level. Our findings showed that the correlation was not very high with r = 0.46. We think that we lacked the amount of data to accurately classify the social activeness of the person. Therefore, in our future work, we are planning to use voice features (pitch, frequency of voice, speaking rate) of microphone, which may help to know the frequency of real-life communication with people, and discovery of Bluetooth devices to identify number of surrounding people and apply machine learning algorithm with these features to better characterize social activity level.

## 7. Conclusions

In this work, we created a group classification system using accurate passive sensing data, to help depressed patients to know their depression severity in a very short period of time. To achieve this, we proposed STDD, a framework that can track five factors influencing depression and perform group classification based on the passive sensing information. This study with 20 participants verified the feasibility of a group classification model with the PHQ-9 [14] as a commonly served ground truth. We conducted the pilot study that made solid contributions to this field, but it revealed some more directions in which work is needed to prove the practicability of such a framework. First, to extract a generalized model for the four-group classification, it is essential to increase the number of participants (to more than 30) in each group and use distinct.

## Figures and Tables

**Table 1:** Literature review: depression tracking with passive sensing.

Applications	User	Purpose	Methods & Data	Passive Sensing	EMA	Interventions
StudentLife [7]	Students	Mental Health & Academic- performance Prediction	Auto-report (activity, mobility, body, sleep, social) Self-report (PHQ-9, stress, self-perceived success, lonliness)	Yes	Yes	No
eMate EMA [8]	Students	Emotion prediction ability measurement	Self-report (emotion / five times a day)	No	Yes	No
iYouUV [9]	Students	Research	Auto-report (social activity, calls, SMS, App use pattern, screen-release, pictures)	Yes	No	No
Headgear [10]	Employee	Depression & Anxiety Detection	Self-report (emotion)	No	Yes	Yes
Socialise [11]	Ordinary	Depression & Anxiety Detection	Auto-report (Bluetooth, GPS, battery status)	Yes	No	No
Mindgauge [12]	Ordinary	Monitoring	Self-report (psychological problems, well-being, resilience)	No	Yes	Yes
Purple robot [16]	Depressive	Depression Detection	Auto-report (physical activity, social activity) Self-report (PHQ-9)	Yes	Yes	No
FINE [17]	Depressive	Depression Detection	Auto-report (smartphone use, social activity, movement) Self-report (emotion, PHQ-9)	Yes	Yes	No
Mobilyze! [5]	Depressive	Prediction & Intervention of Depression	Auto-report (physical activity, social activity) Self-report (emotion)	Yes	Yes	Yes
iHOPE [18]	Depressive	Research for EMA (feasibility & validity)	Auto-report (smartphone usage patterns) Self-report (emotion)	Yes	Yes	Yes
PRIORI [20]	Bipolar	Selection of Risk groups	Auto-report (voice pattern analysis)	Yes	No	No
MONARCA [19,21]	Bipolar	Symptom management & Intervention	Auto-report (accelerometer, call logs, screen on/off time, app usage, browsing history) Self-report (mood, sleep)	Yes	Yes	Yes
Moodrhythm [22]	Bipolar	Monitoring & Intervention	Auto-report (sleep, physical, social activity)	Yes	No	Yes
SIMPle 1.0 [23]	Bioplar	Symptom management & Psycho- educational Intervention	Auto-report (smartphone or SNS time, calls, and physical activity) Self-report (mood, suicidal thoughts)	Yes	Yes	Yes
iBobbly [24]	Depressive	Suicide Prevention	Self-report (emotion, function)	No	Yes	Yes

**Table 2:** List of sensors and features for physical activity and mood level classification.

Symptom Cluster	Sensors	Features
Physical activity	Accelerometer (for each x, y, z axis)	Mean, standard deviation, maximum, minimum, energy, kurtosis, skewness, root mean square, root sum square, sum, sum of absolute values, mean of absolute values, range, median, upper quartile, lower quartile, and median absolute deviation
	Step detector	Number of steps taken
	Significant motion	Number of significant motion sensor triggers
Mood	Sensors used for physical activity	Features used for physical activity
	HRM	Mean, standard deviation

**Table 3:** EMA response rates.

EMA Check-in Points	Response Rate
7 a.m.	0.38
10 a.m.	0.60
1 p.m.	0.64
4 p.m.	0.61
7 p.m.	0.60
10 p.m.	0.58

**Table 4:** Physical activity and mood level classification results.

Symptom Cluster	Precision (Mean ± SD)	Recall (Mean ± SD)	F- Measure (Mean ± SD)	TP Rate (Mean ± SD)
Physical activity	91.20 ± 4.51%	91.10 ± 4.59%	91.05 ± 4.62%	91.10 ± 4.59%
Mood	91.26 ± 4.43%	90.95 ± 4.57%	91.42 ± 4.89%	91.04 ± 4.55%

**Table 5:** Group classification results.

Group Name	Total Instances	Correctly Classified	Total TP Rate
Normal	150	146	97.33%
Mild	150	147	98.00%
Moderate	150	138	92.00%
Severe	150	145	96.67%
Total number	600	576	96.00%

## References

[1] Kessler R.C., Chiu W.T., Demler O., Walters E.E. (2005). Prevalence, Severity, and Comorbidity of 12-Month DSM-IV Disorders in the National Comorbidity Survey Replication. JAMA Psychiatry.

[2] Simon G.E. (2003). Social and economic burden of mood disorders. Biol. Psychiatry.

[3] Katon W., Ciechanowski P. (2002). Impact of major depression on chronic medical illness. J. Psychosom. Res..

[4] Kasckow J., Zickmund S., Rotondi A., Mrkva A., Gurklis J., Chinman M., Fox L., Loganathan M., Hanusa B., Haas G. (2013). Development of Telehealth Dialogues for Monitoring Suicidal Patients with Schizophrenia: Consumer Feedback. Community Ment. Health J..

[5] Burns M., Begale M., Duffecy J., Gergle D., Karr C.J., Giangrande E., Mohr D.C., Proudfoot J., Dear B. (2011). Harnessing Context Sensing to Develop a Mobile Intervention for Depression. J. Med. Internet Res..

[6] American Psychiatric Association (2013). Diagnostic and Statistical Manual of Mental Disorders: DSM-5.

[7] Wang R., Chen F., Chen Z., Li T., Harari G., Tignor S., Zhou X., Ben-Zeev D., Campbell A.T. Studentlife: Assessing mental health, academic performance and behavioural trends of college students using smartphones. Proceedings of the 2014 ACM International Joint Conference on Pervasive and Ubiquitous Computing, Ser. UbiComp ’14.

[8] Van Breda W., Pastor J., Hoogendoorn M., Ruwaard J., Asselbergs J., Riper H. Exploring and Comparing Machine Learning Approaches for Predicting Mood Over Time. Proceedings of the International Conference on Innovation in Medicine and Healthcare.

[9] Becker D., Bremer V., Funk B., Asselbergs J., Riper H., Ruwaard J. How to predict mood? delving into features of smartphone-based data. Proceedings of the 22nd Americas Conference on Information Systems.

[10] Deady M., Johnston D., Milne D., Glozier N., Peters D., Calvo R.A., Harvey S.B., Bibault J.-E., Wolf M., Picard R. (2018). Preliminary Effectiveness of a Smartphone App to Reduce Depressive Symptoms in the Workplace: Feasibility and Acceptability Study. JMIR mHealth uHealth.

[11] Boonstra T.W., Nicholas J., Wong Q.J.J., Shaw F., Townsend S., Christensen H., Chow P., Pulantara I.W., Mohino-Herranz I. (2018). Using Mobile Phone Sensor Technology for Mental Health Research: Integrated Analysis to Identify Hidden Challenges and Potential Solutions. J. Med. Internet Res..

[12] Choi I., Milne D., Deady M., Calvo R.A., Harvey S.B., Glozier N., Calear A., Yeager C., Musiat P., Torous J. (2018). Impact of Mental Health Screening on Promoting Immediate Online Help-Seeking: Randomized Trial Comparing Normative Versus Humor-Driven Feedback. JMIR Ment. Health.

[13] Crosby R.D., Lavender J.M., Engel S.G., Wonderlich S.A. (2016). Ecological Momentary Assessment.

[14] Kroenke K., Spitzer R.L., Williams J.B. (2001). The PHQ-9: Validity of a brief depression severity measure. J. Gen. Intern. Med..

[15] Saeb S., Zhang M., Karr C.J., Schueller S.M., Corden M.E., Kording K.P., Mohr D.C. (2015). Mobile phone sensor correlates of depressive symptom severity in daily-life behaviour: An exploratory study. J. Med. Internet Res..

[16] Schueller S.M., Begale M., Penedo F.J., Mohr D.C. (2014). Purple: A modular system for developing and deploying behavioural intervention technologies. J. Med. Internet Res..

[17] Dang M., Mielke C., Diehl A., Haux R. (2016). Accompanying Depression with FINE - A Smartphone-Based Approach. Stud. Health Technol. Inform..

[18] Hung S., Li M.-S., Chen Y.-L., Chiang J.-H., Chen Y.-Y., Hung G.C.-L. (2016). Smartphone-based ecological momentary assessment for chinese patients with depression: An exploratory study in Taiwan. Asian J. Psychiatry.

[19] Bardram J.E., Frost M., Szántó K., Faurholt-Jepsen M., Vinberg M., Kessing L.V. Designing mobile health technology for bipolar disorder: A field trial of the Monarca system. Proceedings of the SIGCHI Conference on Human Factors in Computing Systems, Ser. CHI ’13.

[20] Gideon J., Provost E., McInnis M. Mood state prediction from speech of varying acoustic quality for individuals with bipolar disorder. Proceedings of the 2016 IEEE International Conference on Acoustics, Speech and Signal Processing (ICASSP).

[21] Frost M., Doryab A., Faurholt-Jepsen M., Kessing L.V., Bardram J.E. Supporting disease insight through data analysis. Proceedings of the 2013 ACM International Joint Conference on Pervasive and Ubiquitous Computing (UbiComp 2013).

[22] Voida S., Matthews M., Abdullah S., Xi M.C., Green M., Jang W.J., Hu D., Weinrich J., Patil P., Rabbi M. Moodrhythm: Tracking and supporting daily rhythms. Proceedings of the 2013 ACM Conference on Pervasive and Ubiquitous Computing Adjunct Publication, Ser. UbiComp ’13 Adjunct.

[23] Hidalgo-Mazzei D., Mateu A., Reinares M., Undurraga J., del Mar Bonnín C., Sánchez-Moreno J., Vieta E., Colom F. (2015). Self-monitoring and psychoeducation in bipolar patients with a smart-phone application (sim-ple) project: Design, development and studies protocols. BMC Psychiatry.

[24] Tighe J., Shand F., Ridani R., MacKinnon A., De La Mata N.L., Christensen H. (2017). Ibobbly mobile health intervention for suicide prevention in Australian Indigenous youth: A pilot randomised controlled trial. BMJ Open.

[25] Qualtrics. https://www.qualtrics.com/.

[26] Upton J., Gellman M.D., Turner J.R. (2013). Beck Depression Inventory (BDI). Encyclopedia of Behavioral Medicine.

[27] State-Trait Anxiety Inventory (STAI). https://www.apa.org/pi/about/publications/caregivers/practice-settings/assessment/tools/trait-state.

[28] Pollack M. (2005). Comorbid anxiety and depression. J. Clin. Psychiatry.

[29] Mikolajczyk R., El Ansari W., Maxwell A. (2009). Food consumption frequency and perceived stress and depressive symptoms among students in three European countries. Nutr. J..

[30] Wallin M.S., Rissanen A.M. (1994). Food and mood: Relationship between food, serotonin and affective disorders. Acta Psychiatr. Scand..

[31] Manea L., Gilbody S., McMillan D. (2011). Optimal cut-off score for diagnosing depression with the Patient Health Questionnaire (PHQ-9): A meta-analysis. Can. Med. Assoc. J..

[32] Kocalevent R.D., Hinz A., Brahler E. (2013). Standardization of the depression screener Patient Health Questionnaire (PHQ-9) in the general population. Gen. Hosp. Psychiatry.

[33] Manea L., Gilbody S., McMillan D. (2015). A diagnostic meta-analysis of the Patient Health Questionnaire-9 (PHQ-9) algorithm scoring method as a screen for depression. Gen. Hosp. Psychiatry.

[34] Django. https://www.djangoproject.com/.

[35] Hall M., Frank E., Holmes G., Pfahringer B., Reutemann P., Witten I.H. (2009). The WEKA data mining software. ACM SIGKDD Explor. Newsl..

[36] Quiroz J.C., Yong M.H., Geangu E. Emotion recognition using smart watch accelerometer data: Preliminary findings. Proceedings of the 2017 ACM International Joint Conference on Pervasive and Ubiquitous Computing and Proceedings of the 2017 ACM International Symposium on Wearable Computers, Ser. UbiComp ’17.

[37] Quiroz J.C., Geangu E., Yong M.H., Lee U., Snider D. (2018). Emotion Recognition Using Smart Watch Sensor Data: Mixed-Design Study. JMIR Ment. Health.

[38] KakaoTalk. https://www.kakaocorp.com/service/KakaoTalk?lang=en.

[39] Canzian L., Musolesi M. Trajectories of depression: Unobtrusive monitoring of depressive states by means of smartphone mobility traces analysis. Proceedings of the 2015 ACM International Joint Conference on Pervasive and Ubiquitous Computing, ser. UbiComp ’15.

